# What are the determinants of childhood infections in India’s peri-urban slums? A case study of eight cities

**DOI:** 10.1371/journal.pone.0257797

**Published:** 2021-10-15

**Authors:** Yebeen Ysabelle Boo, Kritika Rai, Meghan A. Cupp, Monica Lakhanpaul, Pam Factor-Litvak, Priti Parikh, Rajmohan Panda, Logan Manikam, On Behalf of the Childhood Infections & Pollution (CHIP) Consortium

**Affiliations:** 1 Nuffield Department of Population Health, University of Oxford, Oxford, United Kingdom; 2 Aceso Global Health Consultants PTE Ltd., Singapore, Singapore; 3 Population, Policy and Practice, UCL Great Ormond Street Institute of Child Health, London, United Kingdom; 4 Brown University School of Public Health, Providence, Rhode Island, United States of America; 5 Whittington Health NHS Trust, London, United Kingdom; 6 Columbia University Mailman School of Public Health, New York, New York, United States of America; 7 Engineering for International Development Centre, Bartlett School of Construction and Project Management, Faculty of Built Environment, University College London, London, United Kingdom; 8 The Public Health Foundation of India, Delhi, India; 9 Department of Epidemiology and Public Health, University College London Institute of Epidemiology and Health Care, London, United Kingdom; International Centre for Diarrhoeal Disease Research, BANGLADESH

## Abstract

**Background:**

Respiratory Tract Infections (RTIs) and Gastro-Intestinal (GI) infections are the leading causes of child mortality and morbidity. This study investigates the associations between the individual, household and slum-level determinants of children’s health and vulnerability to RTIs and GI infections in peri-urban slums in India; an area of research interest at the Childhood Infections and Pollution Consortium.

**Methods:**

The 2015–16 Indian National Family Health Survey was used for data analysis on children aged 0–5 years. NFHS-4 includes data on slums in eight Indian cities, including Delhi, Meerut, Kolkata, Indore, Mumbai, Nagpur, Hyderabad, Chennai. The outcome variables, having *fever and cough (FeCo)* and *diarrhoea* in the last two weeks, were used to define the phenotype of infections; for this analysis fever and cough were measures of RTIs and diarrhoea was used to measure GI infections. Exposures considered in this study include variables at the individual, household and slum level and were all informed by existing literature. Multilevel models were used to estimate the association between exposures and outcomes variables; a prior of Cauchy distribution with a scale of 2.5 was selected when building the multilevel logistic models.

**Results:**

The total sample size of the number of children included in the analysis was *n = 1*,*424*. Data was imputed to account for missingness, and the original and imputed sample showing similar distributions. Results showed that diarrhoea and FeCo were both found to be more present in younger children than older children by a few months. In fixed effects, the odds of developing *FeCo* were higher if the mother perceives the child was born *smaller than average* (AOR 4.41, 1.13–17.17, P<0.05) at individual level. On the other hand, the odds of the diarrhoea outcome were lower if the child was *older* (AOR 0.97, 0.96–0.98, P<0.05) at individual level, and household’s water source was *public tap or standpipe* (AOR 0.54, 0.31–0.96, P<0.05) at household level.

**Conclusion:**

The determinants of health, both social and related to health care, at all levels demonstrated linkages to child morbidity in RTIs and GI infections. The empirical evidence highlights the need for contextualised ideas at each level, including one health approach when designing interventions to improve child health.

## Introduction

**Fig 1:**
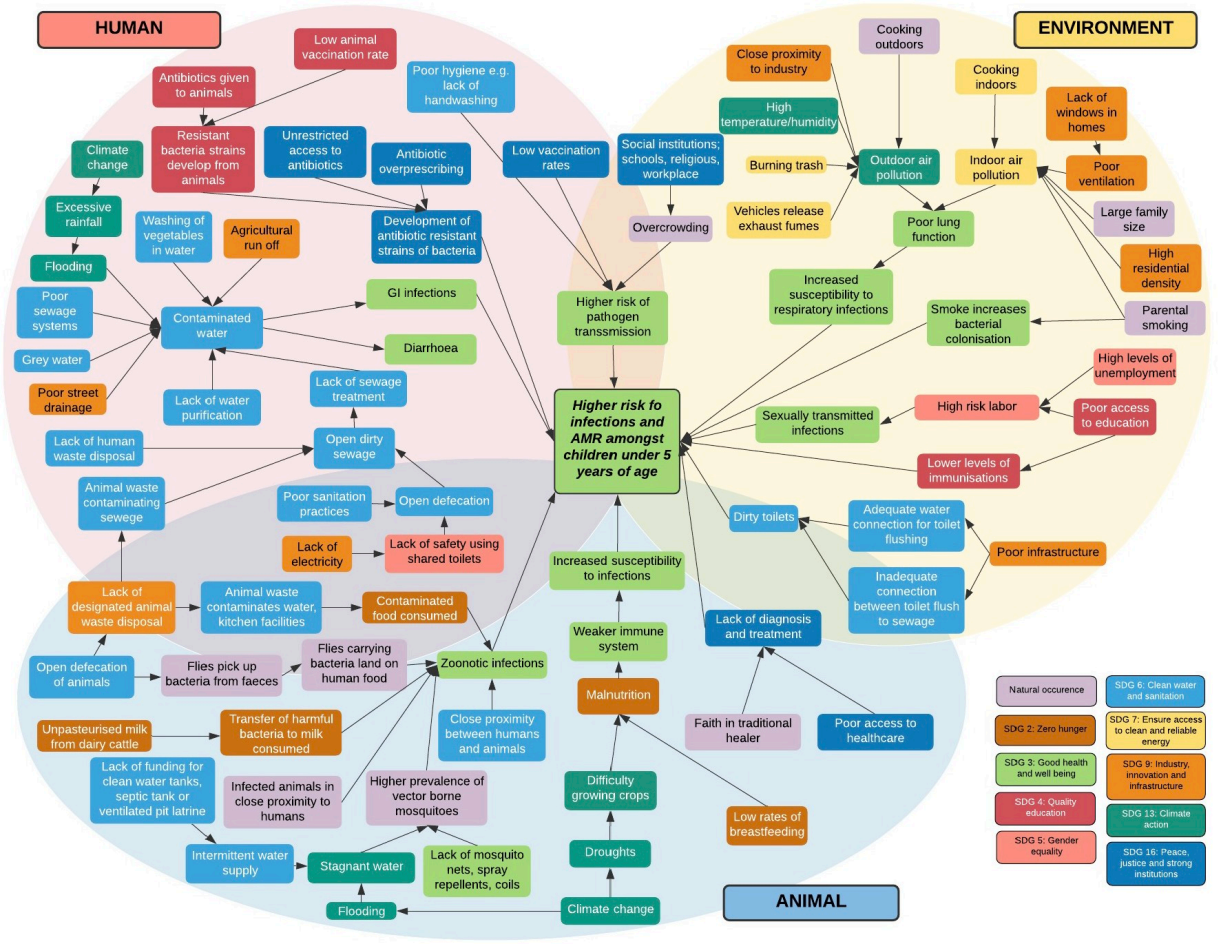
A conceptual map of one health factors associated with infections and AMR in U5s living in urban slums [3].

Reducing child mortality is one of the main aims in achieving Sustainable Development Goal 3: To ensure healthy lives and promote well-being for all at all ages [1]. This is an important task for India in particular, a rapidly growing country, which in 2015, saw the highest rate of child mortality with 1.2 million deaths occurring in under-5 (U5) children (47.8 per 1000 live births). Infectious diseases, particularly Respiratory Tract Infections (RTIs) and Gastro-Intestinal (GI) infections are the leading cause of child mortality in India, with disparities in child mortality across both states and regions [2]. More than nine percent of all U5 mortality in 2015 was due to diarrhoea, and 15.9% was due to pneumonia. These figures indicate that understanding the current infection pathways are important to design interventions for decreasing morbidity and mortality from these infections. This includes tackling a complex network of multiple factors, including the built environment, nutrition, Water and Sanitation Hygiene (WASH), animal husbandry practices, vector control open defecation, and air and water pollution due to open defecation and drainage. As children’s health is intertwined in a complex network between people, animals and their environment (Fig 1), it is important to consider the One Health paradigm. This is particularly vital in the current climate, whereby the burdens of emerging infections as well as traditional infection co-exist.

As per 2011 census data, 30.8% of India’s total population fall between 0 and 15 years of age, while 26.5% of its population live in urban areas. With the rapid, unchecked increase in rural-urban migration, it is now estimated that between 11.2 and 21.1% of India’s urban population are currently living in slums, with a significant portion of this population being children as reflected in India’s total youth population [4]. The UN-Habitat currently defines slums or informal settlements as following [5, 6]:

*“household[s] where the inhabitants suffer one or more of the following ‘household deprivations’: 1) Lack of access to improved water source, 2) Lack of access to improved sanitation facilities, 3) Lack of sufficient living area, 4) Lack of housing durability and, 5) Lack of security of tenure, 6) Lack of separate space for animals, 7)Lack of separate space for cooking, 8)Lack of open space for recreation/playing).”*

Children living in slums are not only exposed to the environmental hazards of disease-causing bacteria, viruses and parasites, but the health effects of these infections are exacerbated by the social and environmental determinants driving health inequalities [7, 8]. For example, the living environments of U5 children often involve high levels of pollution and antimicrobial resistance compared to non-slums; slum environments suffer from severe urban poverty and lack of basic infrastructure [9]. As life course epidemiology posits that childhood exposures are detrimental to later life health increased morbidity, decreased socio-economic positions and higher mortality [10], understanding these variables in slums has significant public health importance.

Despite the strength of evidence for the link between the social and environmental determinants of children’s health and vulnerability to RTIs and GI infections, this association has not been explored intensively in peri-urban slum settings in India [11–14].

This study aims to take an in-depth look at social determinants of health in slums, its effect on the risk of RTIs and GIs in children, which are the leading causes of child mortality in India. This research builds on ongoing work by the Childhood Infections and Pollution Study (CHIP), which aims to reduce infections and antimicrobial resistance in U5 urban slum populations through low-cost behaviour change and slum-upgrading interventions using One health and technology enabled citizen science approaches.

Moreover, this study will aim to explore the social and environmental determinants of children’s health in peri-urban slums in India, using a multilevel logistic regression analysis of child-, household-, and slum-level determinants associated with phenotypic symptoms of RTIs and GI infections in U5 children. Identified protective or risk factors will also be used to inform the explanatory variables of CHIP formative research, from theoretical deduction. This study is innovative in its holistic view of environmental, socio-economic and biological factors of children’s health to examine how different factors simultaneously influence each other and determine outcomes of *fever and cough* and *diarrhoea*.

## Methods

### Dataset

This study uses the 2015–2016 National Family Health Survey, India (NFHS-4), this survey is conducted under the Indian Ministry of Health and Family Welfare to date. NFHS-4 includes data on slums in eight Indian cities, including Delhi, Meerut, Kolkata, Indore, Mumbai, Nagpur, Hyderabad, Chennai [15]. The details of the survey methodology can be reviewed at DHS Methodological Reports No. 26 [16].

We included households if: they had children aged five or under and if they were within a cluster defined as “slum designation by observation”. The identification of slum and non-slum cluster was only conducted in eight Indian cities when administering NFHS-4, therefore, we could not select any children’s data outside of these eight cities. To minimise selection bias, we included all available children’s data that met our criteria collected during NFHS-4. Some cities in this dataset are defined as ‘tier 1’ in size, with a population 100,000 and above (Delhi, Kolkata, Mumbai, Hyderabad, Chennai) while some are ‘tier 2’ with a population 50,000 to 99,999 (Meerut, Indore, Nagpur); for we control for random effects in regression modelling.

### Phenotype

As clinical or laboratory confirmed diagnosis of RTIs or GI infections at a population level is extremely difficult, self-reported responses from structured surveys can be used as a way of phenotyping these infections. To robustly phenotype RTIs and GI infections, this study uses phenotypes derived from previous published studies and clinician input. Three outcome variables were carefully chosen after reviewing a similar peer-reviewed article that used 1998–1999 NFHS data to “understand determinants of health services utilization for children suffering from diarrhoea and respiratory illness in rural Bihar” [17] and expert advice from by Dr Manikam (last author), a senior doctor at Public Health England with paediatric training.

### Model and variable specification

*Outcome variables* are binary in nature and were defined as 0 for No and 1 for Yes, for this analysis fever and cough were measures of RTIs and diarrhoea was used to measure GI infections.

1. A child had both fever and cough in the last two weeks (recoded using: had a fever in the last two weeks and had a cough in the last two weeks). Hereby onwards referred to as *FeCo*.
2. A child had diarrhoea in the last two weeks. Hereby onwards referred to as diarrhoea.

**Fig 2:**
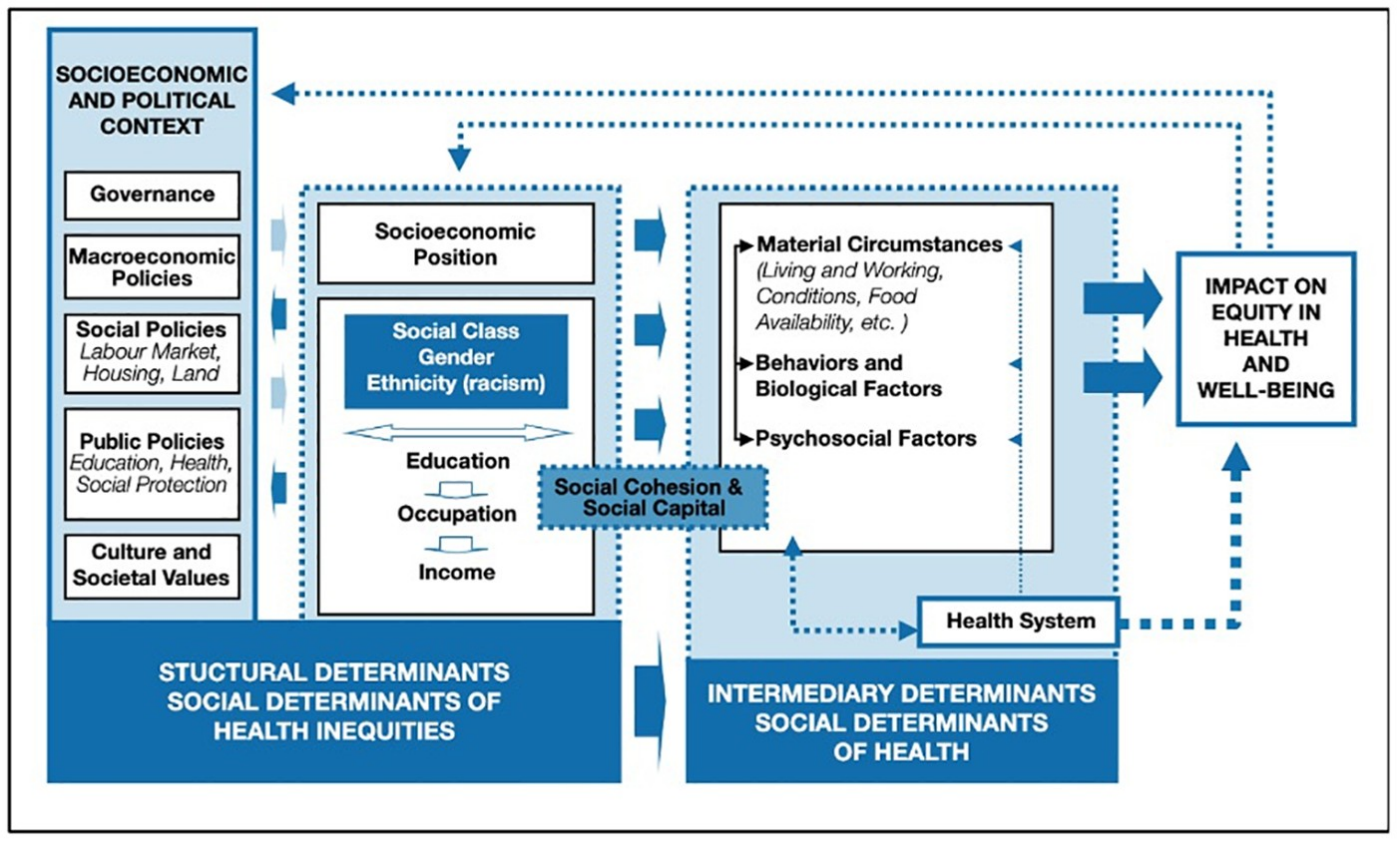
A conceptual framework for action on the social determinants of health [20].

Multilevel models were used in the analyses the consider the association between exposures and outcomes variables; a prior of Cauchy distribution with a scale of 2.5 was selected when building the multilevel logistic models. Exposures were categorised into three levels: individual, household, and slum level [18, 19] as per the World Health Organisation (WHO) Social Determinants of Health Framework (Fig 2), that both structural and intermediary determinants of health, with structural determinants influencing the health inequalities in the wider society [20].

Explanatory variables informed by existing literature (selection rationale for individual and household level variables are explained in further detail in Tables 1 and 2 in the S1 File):

1. Individual level: Sex of child; Child’s age in months, Birthweight (mother’s perception of child’s size at birth); The month of the interview; Never breastfed.
  *The sex of the child* was included as a variable due to sex-differences in virulence of infections being observed in epidemiological studies. *The child’s age* was also included in the analysis as it has been observed that older children have more mature immune systems to fight off pathogens [21, 22]. Next, *Birthweight (mother’s perception)* was also used as epidemiological studies point towards children with low-birthweight having an increased risk of contracting RTIs and GI infections. Since there is a significant incidence of home delivery (M15), a mother’s opinion of the birthweight was used as a variable instead of the actual birth weight [23, 24]. *The month of the interview* was also used to account for how India faces heavy rainfall during the Monsoon season, which has associations with RTIs and GI infections if there are inadequate sanitation or water sources (related to slum environment) [25–27]. Finally, at the individual level, *never breastfed* was used as a variable in the analysis to account for the difference in immunity of breastfed children and non-breastfed children as observed in epidemiological studies [28].
2. Household level: Highest year of maternal education attained; Source of drinking water; Type of toilet facility; Wealth index (a composite score with various ownership overlapping with other household variables listed here as calculated by the NFHS dataset [15]; household has mosquito bed net for sleeping in the household; covered by health insurance; main material of floor; wall; roof; Religion; Caste or tribe; type of cooking fuel; maternal smoking and current marital status of the mother.
  *Highest year of education of a mother* was included in the analysis as epidemiological studies point towards positive effects of maternal education on childhood health outcomes [29, 30]. *Source of drinking water* was also added in the analysis as different levels of contaminants (linked to infections) are found in different water sources in LMIC urban slums [31]. *Type of toilet facility* was also included in this study as previous studies has shown how it has been linked to health and social benefits in LMIC [32]. Furthermore, the *Wealth Index* was used to present a composite measure of a household’s cumulative living standard; this includes the following variables: *household has electricity; radio; television; refrigerator; telephone (landline); bicycle; motorcycle/scooter; car/truck; mosquito bed net for sleeping in the household; covered by health insurance*. Next, *the main material of floor wall and roof* was used to account for the findings of associations between housing materials on children’s health [33, 34]. Type of cooking fuel was also included in the analysis to account for its associations with children’s respiratory infections [35]. Additionally, *Religion; Caste or Tribe* was included so as to account for possible associations of health outcomes with Scheduled Caste status (that play an important role in social determinants in India) [36, 37]. Finally, the *marital status of the mother* was chosen for the analysis to observe any associations between single motherhood and vulnerability in children as observed in previous studies [38].
3. Slum (cluster) level: State; The aggregated mean of years of maternal education attained; wealth index; perceived distance to health facilities. These aggregated means were calculated by slum cluster.

### Missing data & imputation

In the dataset, missing data was observed in ten variables. We chose not to do complete case analysis as the potential for bias is present.

Hot-deck imputation [39] was used for the variables where 50% of observations or more were listed as no answer or not applicable (NA) to create the final dataset. After the imputation, the variance was inflated to account for the imputation method.

### Statistical methods

The units of analysis were U5 children, households within a cluster defined as “slum designation by observation” and slum clusters. Slum-level variables were created by aggregating the mean of the respondents’ characteristics within the NFHS-4 clusters. Data analysis was carried out using R Studio 1.2.5033 and R 3.6.3.

First, the descriptive analysis was completed for each variable of interest and the gathered data were used to inform the best statistical modelling method according to the data. Descriptive analysis revealed that the dataset contained a low prevalence of the outcomes of *FeCo* and *diarrhoea* at only 6% and 11%, respectively. Therefore, variables that did not have enough data to compute meaningful coefficient estimates were removed from the final model. The Adjusted Odds Ratios (AORs) were computed via coefficients obtained from a Bayesian generalised linear model (individual- and household-level) and a random-effects Bayesian generalised linear model (slum-level). The random effects were selected due to determinants at an individual, household and societal level; to reflect this, cluster ID was used to control its effects. To address the two-level hierarchical structure with individual- and household- levels (level 1) embedded within each cluster (level 2), two models were fitted for each outcome. To justify the use of random-effects modelling at the cluster level, variance was measured against the cluster ID. For both outcomes, a variance of 0.4% (with a standard deviation of 0.03) was observed.

- Model 1 fits a non-hierarchical logistic regression of the *FeCo* binary outcome using a weakly informative default prior distribution setting built by Gelman *et al*., which gives a better model when there is separation in the logistic regression [40]. The default setting (Cauchy distribution with scale of 2.5) and *bayesglm* function of the *arm* package were explained in detail in Gelman’s book and was deemed most appropriate for this study’s statistical modelling [41].
- Model 2 fits a random-effects (by clusters identified with the cluster id: v001) multilevel logistic regression of the *FeCo* binary outcome with df = 1 (Cauchy distribution) and scale = 2.5 as the prior distribution, using the *brm* function in the *brms* package. This package allows to fit Bayesian generalised multivariate logistic multilevel models using *stan*. Total number of four Markov chains Monte Carlo (MCMC) were applied to fully explore the target posterior distribution.
- Model 3 fits a non-hierarchical logistic regression of the *diarrhoea* binary outcome, the same method as model 1.
- Model 4 fits a random-effects multilevel logistic regression of the *diarrhoea* binary outcome, the same as method as model 2.

### Ethics

A data archivist of The DHS Program authorised the author to access the NFHS India dataset (NFHS is a publicly available dataset on request) for the sole purpose of statistical reporting and analysis and for this registered study.

## Results

The descriptive analysis of all variables is presented in Table 1. Chi-squared was used for categorical variables and Kruskal-Wallis test was used for numeric variables. The total sample size of the number of children involved in the analysis was 1,424; from a total of 259,627 total children in the NFHS-4 and 4,168 U5 population in eight cities (slum and non-slum). The distribution of both our imputed and original sample are shown in Table 1. In comparison with the distribution of individuals with FeCo and Diarrhoea in the original sample, our imputed sample show a similar distribution.

**Table 1:** Descriptive analysis of under-5 children in total slum population of eight Indian cities, NFHS-4, 2015–16.

	Fever and Cough Absent (N = 1347)	Fever and Cough Present (N = 77)	P Value	Diarrhoea Absent (N = 1268)	Diarrhoea Present (N = 156)	P Value	Total (N = 1424)
**Sex**			0.126			0.393	
Male	684 (50.8%)	46 (59.7%)		645 (50.9%)	85 (54.5%)		730 (51.3%)
Female	663 (49.2%)	31 (40.3%)		623 (49.1%)	71 (45.5%)		694 (48.7%)
**Child’s Age in Months**			0.044			<0.001	
Mean (SD)	29 (17)	25 (15)		30 (17)	22 (15)		29 (16.896)
Median (Q1, Q3)	30 (15, 44)	23 (15, 37)		31 (16, 45)	20 (9, 33)		29 (15, 44)
Min—Max	0–59	2–58		0–59	0–59		0–59
**Size of Child at Birth**			0.078			<0.001	
Very Large	103 (7.6%)	1 (1.3%)		98 (7.7%)	6 (3.8%)		104 (7.3%)
Larger Than Average	178 (13.2%)	8 (10.4%)		172 (13.6%)	14 (9.0%)		186 (13.1%)
Average	909 (67.5%)	53 (68.8%)		865 (68.2%)	97 (62.2%)		962 (67.6%)
Smaller Than Average	102 (7.6%)	9 (11.7%)		89 (7.0%)	22 (14.1%)		111 (7.8%)
Very Small	55 (4.1%)	6 (7.8%)		44 (3.5%)	17 (10.9%)		61 (4.3%)
**Month of Interview**			0.032			<0.001	
March	25 (1.9%)	1 (1.3%)		26 (2.1%)	0 (0.0%)		26 (1.8%)
April	372 (27.6%)	11 (14.3%)		362 (28.5%)	21 (13.5%)		383 (26.9%)
May	588 (43.7%)	33 (42.9%)		564 (44.5%)	57 (36.5%)		621 (43.6%)
June	295 (21.9%)	27 (35.1%)		251 (19.8%)	71 (45.5%)		322 (22.6%)
July	12 (0.9%)	0 (0.0%)		9 (0.7%)	3 (1.9%)		12 (0.8%)
Sept	55 (4.1%)	5 (6.5%)		56 (4.4%)	4 (2.6%)		60 (4.2%)
**State**			<0.001			<0.001	
Madhya Pradesh	306 (22.7%)	3 (3.9%)		294 (23.2%)	15 (9.6%)		309 (21.7%)
Maharashtra	283 (21.0%)	20 (26.0%)		281 (22.2%)	22 (14.1%)		303 (21.3%)
Delhi	110 (8.2%)	4 (5.2%)		101 (8.0%)	13 (8.3%)		114 (8.0%)
Tamil Nadu	104 (7.7%)	5 (6.5%)		99 (7.8%)	10 (6.4%)		109 (7.7%)
Uttar Pradesh	341 (25.3%)	35 (45.5%)		290 (22.9%)	86 (55.1%)		376 (26.4%)
West Bengal	101 (7.5%)	7 (9.1%)		104 (8.2%)	4 (2.6%)		108 (7.6%)
Telangana	102 (7.6%)	3 (3.9%)		99 (7.8%)	6 (3.8%)		105 (7.4%)
**Highest Year of Education of Mother**			0.929			0.915	
Mean (SD)	4.1 (1.6)	4.1 (1.7)		4.1 (1.6)	4.1 (1.7)		4.1 (1.6)
Median (Q1, Q3)	4 (3, 5)	4 (3, 5)		4 (3, 5)	4 (3, 5)		4 (3, 5)
Min—Max	0–7	1–7		0–7	1–7		0–7
**Source of Drinking Water (Accessible in The House or Within Community)**			0.871			0.193	
Piped Water Dwelling	467 (34.7%)	35 (45.5%)		434 (34.2%)	68 (43.6%)		502 (35.3%)
Piped Water Yard or Plot	282 (20.9%)	13 (16.9%)		261 (20.6%)	34 (21.8%)		295 (20.7%)
Public Tap or Standpipe	290 (21.5%)	13 (16.9%)		284 (22.4%)	19 (12.2%)		303 (21.3%)
Tube Well Water	214 (15.9%)	13 (16.9%)		204 (16.1%)	23 (14.7%)		227 (15.9%)
Dug Well: Open or Protected	3 (0.2%)	0 (0.0%)		3 (0.2%)	0 (0.0%)		3 (0.2%)
Surface Water	2 (0.1%)	0 (0.0%)		2 (0.2%)	0 (0.0%)		2 (0.1%)
Rainwater	2 (0.1%)	0 (0.0%)		2 (0.2%)	0 (0.0%)		2 (0.1%)
Tanker Truck	35 (2.6%)	1 (1.3%)		31 (2.4%)	5 (3.2%)		36 (2.5%)
Cart with Small Tank	4 (0.3%)	0 (0.0%)		3 (0.2%)	1 (0.6%)		4 (0.3%)
Bottled Water	42 (3.1%)	2 (2.6%)		38 (3.0%)	6 (3.8%)		44 (3.1%)
Community RO Plant	6 (0.4%)	0 (0.0%)		6 (0.5%)	0 (0.0%)		6 (0.4%)
**Type of Toilet Facility (Accessible in The House or Within Community)**			0.564			0.186	
Flush Toilet	1170 (86.9%)	72 (93.5%)		1096 (86.4%)	146 (93.6%)		1242 (87.2%)
Pit Toilet Latrine	47 (3.5%)	2 (2.6%)		47 (3.7%)	2 (1.3%)		49 (3.4%)
No Facility	109 (8.1%)	2 (2.6%)		103 (8.1%)	8 (5.1%)		111 (7.8%)
Composting Toilet	4 (0.3%)	0 (0.0%)		4 (0.3%)	0 (0.0%)		4 (0.3%)
Dry Toilet	4 (0.3%)	0 (0.0%)		4 (0.3%)	0 (0.0%)		4 (0.3%)
Other	13 (1.0%)	1 (1.3%)		14 (1.1%)	0 (0.0%)		14 (1.0%)
**Main Floor Material**			0.415			0.755	
Natural	84 (6.2%)	6 (7.8%)		81 (6.4%)	9 (5.8%)		90 (6.3%)
Rudimentary	81 (6.0%)	8 (10.4%)		82 (6.5%)	7 (4.5%)		89 (6.2%)
Finished	1181 (87.7%)	63 (81.8%)		1104 (87.1%)	140 (89.7%)		1244 (87.4%)
Other	1 (0.1%)	0 (0.0%)		1 (0.1%)	0 (0.0%)		1 (0.1%)
**Main Wall Material**			0.546			0.407	
Natural	60 (4.5%)	2 (2.6%)		59 (4.7%)	3 (1.9%)		62 (4.4%)
Rudimentary	26 (1.9%)	3 (3.9%)		26 (2.1%)	3 (1.9%)		29 (2.0%)
Finished	1258 (93.4%)	72 (93.5%)		1180 (93.1%)	150 (96.2%)		1330 (93.4%)
Other	3 (0.2%)	0 (0.0%)		3 (0.2%)	0 (0.0%)		3 (0.2%)
**Main Roof Material**			0.436			0.292	
Natural	41 (3.0%)	0 (0.0%)		39 (3.1%)	2 (1.3%)		41 (2.9%)
Rudimentary	32 (2.4%)	2 (2.6%)		28 (2.2%)	6 (3.8%)		34 (2.4%)
Finished	1269 (94.2%)	75 (97.4%)		1196 (94.3%)	148 (94.9%)		1344 (94.4%)
Other	5 (0.4%)	0 (0.0%)		5 (0.4%)	0 (0.0%)		5 (0.4%)
**Religion**			0.121			<0.001	
Hindu	795 (59.0%)	35 (45.5%)		764 (60.3%)	66 (42.3%)		830 (58.3%)
Muslim	521 (38.7%)	41 (53.2%)		478 (37.7%)	84 (53.8%)		562 (39.5%)
Christian	6 (0.4%)	1 (1.3%)		7 (0.6%)	0 (0.0%)		7 (0.5%)
Sikh	2 (0.1%)	0 (0.0%)		1 (0.1%)	1 (0.6%)		2 (0.1%)
Buddhist/Neo-Buddhist	20 (1.5%)	0 (0.0%)		15 (1.2%)	5 (3.2%)		20 (1.4%)
Jain	3 (0.2%)	0 (0.0%)		3 (0.2%)	0 (0.0%)		3 (0.2%)
**Caste or Tribe**			0.489			0.533	
Caste	1302 (96.7%)	74 (96.1%)		1224 (96.5%)	152 (97.4%)		1376 (96.6%)
Tribe	13 (1.0%)	0 (0.0%)		11 (0.9%)	2 (1.3%)		13 (0.9%)
No Caste or Tribe	32 (2.4%)	3 (3.9%)		33 (2.6%)	2 (1.3%)		35 (2.5%)
**Number of Household Members**			0.062			0.233	
Mean (SD)	6.609 (3.202)	7.273 (3.401)		6.603 (3.183)	6.981 (3.463)		6.645 (3.215)
Median (Q1, Q3)	6 (4, 8)	7 (5, 9)		6 (4, 8)	6 (4, 8)		6 (4, 8)
Min—Max	1–21	3–18		1–21	3–21		1–21
**Type of Cooking Fuel**			0.271			0.031	
Electricity	21 (1.6%)	0 (0.0%)		17 (1.3%)	4 (2.6%)		21 (1.5%)
LPG, Natural Gas	1035 (76.8%)	63 (81.8%)		977 (77.1%)	121 (77.6%)		1098 (77.1%)
Biogas	5 (0.4%)	0 (0.0%)		5 (0.4%)	0 (0.0%)		5 (0.4%)
Kerosene	98 (7.3%)	2 (2.6%)		95 (7.5%)	5 (3.2%)		100 (7.0%)
Coal, Lignite	5 (0.4%)	0 (0.0%)		4 (0.3%)	1 (0.6%)		5 (0.4%)
Charcoal	7 (0.5%)	0 (0.0%)		6 (0.5%)	1 (0.6%)		7 (0.5%)
Wood	114 (8.5%)	5 (6.5%)		111 (8.8%)	8 (5.1%)		119 (8.4%)
Straw/Shrubs/Grass	4 (0.3%)	0 (0.0%)		3 (0.2%)	1 (0.6%)		4 (0.3%)
Animal Dung	55 (4.1%)	6 (7.8%)		47 (3.7%)	14 (9.0%)		61 (4.3%)
No Food Cooked in House	1 (0.1%)	0 (0.0%)		1 (0.1%)	0 (0.0%)		1 (0.1%)
Other	2 (0.1%)	1 (1.3%)		2 (0.2%)	1 (0.6%)		3 (0.2%)
**Wealth Index**			0.029			0.003	
Mean (SD)	3.8 (1.0)	4.1 (0.9)		3.8 (1.0)	4.1 (0.9)		3.8 (1.0)
Median (Q1, Q3)	4 (3, 5)	4 (4, 5)		4 (3, 5)	4 (4, 5)		4 (3, 5)
Min—Max	1–5	1–5		1–5	1–5		1–5
**Have Mosquito Bed Net for Sleeping in The Household**			0.437			0.865	
No	1162 (86.3%)	64 (83.1%)		1091 (86.0%)	135 (86.5%)		1226 (86.1%)
Yes	185 (13.7%)	13 (16.9%)		177 (14.0%)	21 (13.5%)		198 (13.9%)
**Does Not Use Tobacco**			0.522			0.089	
No	36 (2.7%)	3 (3.9%)		38 (3.0%)	1 (0.6%)		39 (2.7%)
Yes, Smokes Nothing	1311 (97.3%)	74 (96.1%)		1230 (97.0%)	155 (99.4%)		1385 (97.3%)
**Covered by Health Insurance**			0.321			0.008	
No	1213 (90.1%)	72 (93.5%)		1135 (89.5%)	150 (96.2%)		1285 (90.2%)
Yes, Covered	134 (9.9%)	5 (6.5%)		133 (10.5%)	6 (3.8%)		139 (9.8%)
**Current Marital Status of Mother**			0.83			0.945	
Never in Union	1 (0.1%)	0 (0.0%)		1 (0.1%)	0 (0.0%)		1 (0.1%)
Married	1334 (99.0%)	76 (98.7%)		1256 (99.1%)	154 (98.7%)		1410 (99.0%)
Widowed	5 (0.4%)	0 (0.0%)		4 (0.3%)	1 (0.6%)		5 (0.4%)
Divorced	1 (0.1%)	0 (0.0%)		1 (0.1%)	0 (0.0%)		1 (0.1%)
Separated	6 (0.4%)	1 (1.3%)		6 (0.5%)	1 (0.6%)		7 (0.5%)
**Perceived Distance to Health Facilities**			0.618			0.026	
No Problem	549 (40.8%)	32 (41.6%)		520 (41.0%)	61 (39.1%)		581 (40.8%)
Not A Big Problem	432 (32.1%)	21 (27.3%)		414 (32.6%)	39 (25.0%)		453 (31.8%)
Big Problem	366 (27.2%)	24 (31.2%)		334 (26.3%)	56 (35.9%)		390 (27.4%)
**Breastfed**			0.866			0.104	
Ever Breastfed	1232 (91.5%)	70 (90.9%)		1154 (91.0%)	148 (94.9%)		1302 (91.4%)
Never Breastfed	115 (8.5%)	7 (9.1%)		114 (9.0%)	8 (5.1%)		122 (8.6%)

Table 1 also shows that both diarrhoea and FeCo were found to be present in younger children more than older children by a few months (mean age 25 months; 29 months respectively and 22 months; 30 months, respectively).

Table 2 represents the AORs (95% confidence interval) of final models 1, 2, 3 and 4. In fixed effects (model 1 and 3), the odds of developing *FeCo* were higher if the mother perceives the child was born *smaller than average* (AOR 4.41, 1.13–17.17). On the other hand, the odds of the diarrhoea outcome were lower if the child was *older* (AOR 0.97, 0.96–0.98) and household’s water source is *public tap or standpipe* (AOR 0.54, 0.31–0.96).

**Table 2:** Adjusted odd ratios (95% CI of all models).

	AOR (95% CI)			
	Model 1	Model 2	Model 3	Model 4
**Sex (Male)**				
Female	0.71 (0.43–1.15)	0.96 (0.82–1.11)	0.91 (0.63–1.31)	0.98 (0.84–1.15)
**Age, Month**	0.99 (0.97–1.00)	1.00 (0.99–1.00)	0.97* (0.96–0.98)	0.99 (0.99–1.00)
**Birthweight (very large)**				
Larger than average	2.43 (0.63–9.28)	1.07 (0.74–1.54)	0.81 (0.31–2.08)	0.96 (0.67–1.38)
Average	2.69 (0.81–8.96)	1.07 (0.78–1.45)	0.95 (0.42–2.15)	0.97 (0.71–1.32)
Smaller than average	4.41* (1.13–17.17)	1.13 (0.75–1.70)	1.78 (0.70–4.55)	1.11 (0.73–1.67)
Very small	3.20 (0.75–13.75)	1.10 (0.68–1.78)	2.43 (0.89–6.65)	1.26 (0.77–2.06)
**Interview Month (March)**				
April	0.74 (0.18–3.09)	1.04 (0.52–2.07)	1.61 (0.39–6.59)	1.39 (0.69–2.81)
May	1.45 (0.37–5.62)	1.00 (0.52–1.93)	2.72 (0.69–10.74)	1.35 (0.69–2.62)
June	1.54 (0.38–6.30)	0.95 (0.47–1.93)	5.90* (1.47–23.65)	1.33 (0.63–2.79)
July	0.41 (0.01–11.42)	0.89 (0.30–2.64)	4.74 (0.67–33.53)	1.45 (0.47–4.43)
Sept	2.69 (0.54–13.36)	1.00 (0.46–2.18)	1.35 (0.26–6.90)	1.26 (0.57–2.81)
**Never breastfed**	1.21 (0.52–2.81)	1.02 (0.77–1.36)	1.02 (0.91–1.14)	1.01 (0.95–1.06)
**Maternal Education, Year**	0.99 (0.85–1.15)	0.99 (0.94–1.04)	1.67 (0.99–2.84)	1.11 (0.87–1.40)
**Water source (Piped water into dwelling)**				
piped water yard or plot	0.77 (0.37–1.59)	0.98 (0.78–1.23)	1.07 (0.57–2.02)	1.02 (0.78–1.33)
public tap or standpipe	1.10 (0.51–2.36)	0.99 (0.76–1.28)	0.54* (0.31–0.96)	0.85 (0.66–1.09)
Tube well water	0.67 (0.32–1.40)	0.95 (0.74–1.23)	0.65 (0.02–27.32)	0.99 (0.15–6.67)
Dug well: open or protected	0.72 (0.01–37.44)	0.92 (0.15–5.80)	0.73 (0.01–36.73)	1.03 (0.12–8.51)
Surface water	0.46 (0.02–14.03)	0.89 (0.11–7.04)	0.71 (0.01–36.32)	0.87 (0.11–7.23)
Rainwater	0.96 (0.01–106.36)	1.12 (0.13–9.42)	2.02 (0.61–6.74)	1.09 (0.61–1.95)
Tanker truck	0.89 (0.14–5.62)	1.04 (0.57–1.88)	3.52 (0.34–36.35)	1.35 (0.32–5.75)
Cart with small tank	0.56 (0.02–18.45)	0.90 (0.22–3.65)	2.29 (0.85–6.17)	1.11 (0.67–1.85)
Bottled water	0.92 (0.22–3.84–1.00)	1.00 (0.59–1.67)	0.39 (0.01–10.09)	0.83 (0.23–3.01)
Community RO plant	0.40 (0.01–10.79)	0.83 (0.23–2.97)	0.52 (0.11–2.43)	0.92 (0.58–1.47)
**Toilet (Flush toilet)**				
Pit toilet latrine	0.99 (0.19–5.10)	0.98 (0.62–1.56)	1.10 (0.47–2.55)	1.04 (0.73–1.47)
No facility	0.38 (0.09–1.55)	0.97 (0.69–1.36)	0.52 (0.02–16.30)	0.90 (0.18–4.60)
Composting toilet	0.68 (0.01–31.65)	0.97 (0.20–4.82)	0.60 (0.02–21.13)	0.97 (0.23–4.14)
Dry toilet	0.61 (0.02–21.52)	0.85 (0.20–3.54)	0.22 (0.01–3.99)	0.83 (0.38–1.81)
Other	2.02 (0.27–15.23)	1.08 (0.49–2.35)	2.09 (0.41–10.58)	1.17 (0.62–2.19)
**Floor (natural)**				
Rudimentary	1.06 (0.30–3.73)	1.04 (0.63–1.72)	0.96 (0.01–106.31)	1.15 (0.07–18.71)
Finished	0.56 (0.19–1.63)	0.93 (0.62–1.40)	1.49 (0.27–8.32)	1.09 (0.54–2.18)
Other	0.82 (0.01–54.91)	0.92 (0.06–14.42)	1.27 (0.33–4.93)	1.02 (0.63–1.67)
**Wall (natural)**				
Rudimentary	3.59 (0.53–24.18)	1.14 (0.57–2.29)	0.77 (0.01–43.32)	1.07 (0.19–5.96)
Finished	0.89 (0.18–4.42)	0.97 (0.60–1.55)	1.72 (0.34–8.66)	1.15 (0.56–2.38)
Other	0.89 (0.01–75.60)	1.00 (0.18–5.40)	0.95 (0.24–3.70)	1.02 (0.60–1.72)
**Roof (natural)**				
Rudimentary	2.44 (0.21–28.20)	1.09 (0.53–2.24)	0.60 (0.02–22.21)	1.12 (0.30–4.20)
Finished	5.00 (0.58–42.82)	1.15 (0.70–1.88)	1.73* (1.12–2.67)	1.10 (0.90–1.34)
Other	0.85 (0.01–62.87)	1.11 (0.28–4.38)	0.27 (0.01–5.93)	0.93 (0.30–2.86)
**Distance to health centre (no problem)**				
Not a big problem	0.74 (0.40–1.34)	0.96 (0.79–1.17)	0.94 (0.31–2.86)	1.03 (0.62–1.71)
Big problem	1.14 (0.61–2.12)	0.97 (0.76–1.24)	0.68 (0.26–1.80)	0.92 (0.51–1.66)
**Cooking fuel (electricity)**				
LPG, natural gas	1.95 (0.43–8.87)	1.09 (0.62–1.91)	0.48 (0.02–14.15)	0.81 (0.19–3.42)
Biogas	0.58 (0.02–20.81)	0.93 (0.21–4.18)	0.56 (0.15–2.04)	0.93 (0.48–1.81)
Kerosene	0.75 (0.12–4.83)	0.99 (0.53–1.88)	2.13 (0.20–22.23)	1.30 (0.32–5.25)
Coal, lignite	0.65 (0.02–27.31)	0.97 (0.25–3.79)	1.11 (0.14–8.90)	1.07 (0.31–3.69)
Charcoal	0.76 (0.01–41.84)	1.00 (0.31–3.25)	0.65 (0.20–2.13)	0.92 (0.48–1.77)
Wood	1.95 (0.37–10.42)	1.14 (0.61–2.14)	1.98 (0.18–21.60)	1.23 (0.26–5.91)
Straw, shrubs, grass	0.76 (0.01–42.20)	1.03 (0.23–4.58)	1.09 (0.34–3.51)	1.03 (0.51–2.08)
Animal dung	3.02 (0.55–16.72)	1.17 (0.60–2.29)	0.54 (0.02–18.75)	0.78 (0.05–12.07)
No food cooked in house	0.75 (0.01–40.64)	0.94 (0.05–16.88)	7.52 (0.31–180.32)	1.57 (0.22–11.21)
Other	20.61 (0.47–901.21)	2.08 (0.29–14.75)	1.18 (0.86–1.64)	1.03 (0.88–1.19)
**Wealth Index (1, poorest-5, wealthiest)**	1.31 (0.84–2.04)	1.02 (0.87–1.19)	1.05 (0.60–1.84)	0.98 (0.76–1.25)
**Mosquito net (do not have a net)**	1.51 (0.76–3.01)	1.01 (0.79–1.29)	2.17 (0.39–12.28)	1.04 (0.65–1.67)
**Mother doesn’t smoke (smokes)**	0.42 (0.11–1.57)	0.88 (0.53–1.44)	0.55 (0.23–1.30)	0.96 (0.73–1.25)
**Health insurance (no insurance)**	0.93 (0.35–2.46)	1.00 (0.76–1.32)	0.74 (0.08–6.90)	1.00 (0.16–6.15)
**State (Madhya Pradesh)**				
Maharashtra		1.16 (0.79–1.69)		1.01 (0.70–1.47)
Delhi		1.14 (0.73–1.78)		1.19 (0.77–1.84)
Tamil Nadu		1.11 (0.72–1.72)		1.10 (0.72–1.68)
Uttar Pradesh		1.18 (0.76–1.86)		1.39 (0.88–2.20)
West Bengal		1.20 (0.79–1.82)		0.97 (0.62–1.50)
Telangana		0.95 (0.63–1.43)		0.89 (0.59–1.36)
**Slum: Wealth Index (1, poorest-5, wealthiest)**		1.05 (0.87–1.27)		1.02 (0.85–1.22)
**Slum: Distance to health centre (no problem)**		1.09 (0.86–1.36)		1.09 (0.87–1.36)
**Slum: Maternal Education, Year**		1.04 (0.92–1.18)		0.97 (0.85–1.10)

AOR: adjusted odd ratios; LCI: lower confidence interval; UCI: upper confidence interval; figures in bold* indicate statistically significant at the p<0.05 level; blue shade: different direction of odd ratios between models.

Although we report on statistically significant finding here, it is noteworthy that many of our variables at individual, slum and household level were not statistically significant in our models.

### Individual level

In model 1 and 2, the child was more protective towards FeCo, if they were born *very large (mother’s perception)*. There were lower odds of reported *FeCo* symptoms in U5, if the interview was conducted in *July* (AOR 2.69, 0.54–13.36).

In models 3 and 4, older children were more protected. For example, for each month of age, the odds of diarrhoea decreased by 3% (AOR 0.97, 0.96–0.98). The odds of diarrhoea were higher when mothers perceived their child’s birth weight as *smaller than average* (AOR 1.78, 0.70–4.55) or *very small* (AOR 2.43, 0.89–6.65) while the odds of diarrhoea were less if the interview was conducted in *March* compared to other months.

### Household level

In model 1 and 2, a one-year increase in the highest level of maternal education showed decreased odds of developing *FeCo* (AOR 0.99, 0.94–1.04). Households using *Piped water into dwelling* were more likely to develop *FeCo* than other sources, except *rainwater* (AOR 1.12, 0.13–9.42) and *tanker trucks* (AOR 1.04, 0.57–1.88). Other sources of water include; *water piped from water yard or plot*, *public taps or standpipes*, *tube well water*, *open or protected dug wells*, *surface water*, *carts with small water tanks*, *bottled water* or a *community Reverse Osmosis (RO) plants*.

Households with a *flush toilet* did not seem to have significant differences in odds when compared to other types of toilets (Flush toilet, Pit toilet latrine, No facility, Composing toilet, and other), having a *dry toilet* showed protective trends against *FeCo* (AOR 0.85, 0.20–3.54). The implications of this result will be further discussed in the Discussion section. The rudimentary material type for wall and floor showed trends of adverse effects for *FeCo*. Among cooking fuel, households using *animal dung* had the highest odds of developing FeCo (AOR 1.17, 0.60–2.29). Lastly, on par with the WHO’s current initiative, children with mothers that do not use *tobacco* had lower odds of developing FeCo (AOR 0.88, 0.53–1.44).

In model 3 and 4, in comparison, households that use *tanker trucks* showed the highest odds of reporting diarrhoea (AOR 1.35, 0.32–5.75). Similar to model 1 and 2, households with a *flush toilet* did not seem to have significant differences in odds when compared to other types of toilets, however, having a *dry toilet* showed protective trends against diarrhoea (AOR 0.83, 0.38–1.81). However, these inferences results will be further explored in the discussion. The finished materials for wall and floors showed adverse associations, in contrast to what we can observe in *FeCo*. This association will be further discussed below. With cooking fuels, *no food cooked in house* (AOR 1.57, 0.22–11.21) were associated with odds of diarrhoea. Although many of the findings lack significance, they are indicative of trends that can be explored by CHIP in future.

### Slum level

As model 2 and 4 suggest, the state *Telangana* was most protected from *FeCo*, while *FeCo* was more prevalent in the slums where the community perceived the greatest distance between their home and the healthcare centre (AOR 1.09, 0.86–1.36). In contrast, *Delhi* and *Uttar Pradesh* were more likely to develop diarrhoea than other states; Uttar Pradesh showed the highest odds of all states, with an AOR of 1.39 (0.88–2.20). Furthermore, slum-level increases in the highest year of maternal education seems to reduce odds of children’s diarrhoeal symptoms by 3% (for every increase in total education year).

## Discussion

The descriptive analysis reflects the challenging socio-economic settings of Indian slums. For example, the mean number of total household members was 6.65 (SD: 3.21), which may indicate a problem of overcrowding as most slum households are unlikely to have sufficient space even if the mean number is similar to non-slum households. Additionally, the mean of the highest year of education completed by mother was 4.1 years out of a possible 15 years.

This study finds that children who are younger in months of age and/or male were discovered to be at higher risk of developing both morbidities; reaffirming results from other epidemiological studies [27–31]. Having very small- or smaller-than-average maternally reported birth weight also increased the odds of both morbidities. This echoes existing findings that highlight associations between low birth weight and increased risk of infections and other aspects of ill health [42]. Low birth weight is also a well-established determinant of child development as well as decreased survival; and plays a major role on the onset of health consequences in an individuals’ life course [43].

Interestingly, the month in which the survey interviews were conducted showed variation in reporting both the morbidities; FeCo was most prevalent in May, whereas diarrhoea was least prevalent in June. This suggests a seasonality in childhood infections, this has previously been observed in past studies whereby infection transmission patterns are impacted by the weather [44]. In addition to seasonal variations, climate conditions and environmental pollutions may also make a contribution to childhood infections [45]. While it would have been beneficial to explore this using multiple year data, it was not feasible to link NFHS-4 individual code to NFHS-3, as many individuals were missing. This was mainly due to the fact that children were not born at the time when NFHS-3 was conducted. Lastly, the odds of being breastfed were protective towards any symptoms of infection, this has been affirmed by past epidemiological studies that show breastfeeding to have several health benefits for children, due to the protective factors in the breast milk against infections [46]. However, it is important to note the lack of precision in the survey instrument; as children who were breastfed at least once were simply recorded as *ever breastfed*, thus there is a possibility in underestimation of association–meaning that the effect of association may have been bigger than presented in our results.

At the household-level, the type of housing material showed distinctive differences, *finished* floor and wall materials showed highly protected trends for *FeCo*. However, the results also showed, that for *diarrhoea*; finished materials for wall and floors showed adverse associations. This finding does not sit in line with other studies conducted, whereby finished flooring showed protective effects against the occurrence of diarrhoea [47]. The difference in results may be due to the possibilities in that these results having uncontrolled confounding. For instance, variables such as hand washing behaviour and proximity to sanitation facilities were not controlled for [48]. This was due to the lack of data availability on these mentioned determinants.

Interestingly, having a *dry toilet* in the household was protective for both morbidities; previous studies surrounding the use of dry toilets have observed a reduced risk of infection transmission due to the waterless mechanism by sealing the faeces through creating a barrier between faeces and the human [49]. This should be further explored in future epidemiological research. The benefits of dry toilets also include: the usage of less water and environmental benefits associated with less contamination of water sources reduction [50]. Thus, dry toilets could be a good policy intervention that is both sustainable towards global water shortage, whilst also affordable to be implemented for slum regeneration. However, we must note that despite the environmental benefits dry toilets deliver, issues concerning social and cultural factors must be considered. Perceptions and prejudices regarding handling excreta that may not provide modest coverage have an important and significant impact on individuals. Additionally, gender issues are frequently discussed in literature surrounding the use of dry toilets. Women use toilets for reasons other than excretion in many geographic locations: for instance, during their menstrual cycles and for child care [51]. Thus, it is important to note that when making suggestions about the use of dry toilets; the issues of inconvenience for women must also be taken into serious consideration.

The results also showed that the use of *tanker trucks for supplying water to the slums* presented an increased odd in some morbidities. However, it must be stated that these results may have been due to the tanker trucks sold in slum environments containing water that was contaminated, as well as further issues with water storage; this includes lack of hygiene upkeep [52, 53]. For instance, in order for the water source to be safe from tanker trucks, the water tank pumps must be rigorously cleaned before use, after major maintenance and at least every 3 months. If this is not completed under a correct cleaning procedure, the chances of clean and safe water from tanker trucks are significantly lowered [52]. Additionally, we are able to assume that this upkeep might not have been regularly maintained; this is due to the prevalence of individuals in our dataset that mention the use of tanker trucks being small (2.5% of those residing in slums). Thus, there is a possibility that the safe delivery and management of tanker trucks were limited and not operationalised to optimise sufficiency as explored by previous studies surrounding water safety and hygiene [52].

In terms of cooking fuels, *animal dung* was associated with higher reporting of FeCo, whereas *no food cooked in house* were adversely associated with diarrhoea. However, this may also be associated with affluence, rather than only the type of fuel used; thus, further research is required. Moreover, children with mothers who do not smoke were protective towards both morbidities, this sits in line with many epidemiological studies whereby maternal smoking is associated with increased risk of infant and childhood disease and mortality (through environmental exposure and the diversion of household income) [54, 55]. Hence encouraging mothers not to smoke could be another area to increase awareness as part of Indian public health campaigns. For both morbidities, it was evident that owning health *insurance* was protective for children, however, higher *wealth index* was only protective towards diarrhoea. This may indicate that U5 children from households that have higher socio-economic status are more likely to get less diarrhoea, whereas *FeCo* could not be a main concern for many households, even if their socio-economic status is higher. This implies that public health campaigns should increase the awareness of the danger of RTIs and potential mortality or morbidity that U5 children can face from infecting them. It is important to note how these morbidities are cared for in each household; whether they sought clinical treatment or home remedies.

At the slum-level, there were clear differences between states in each morbidity, children in *Telangana* were most protective from *FeCo* whereas children in *Delhi* and *Uttar Pradesh* were most likely to develop diarrhoea. This can be explained by state differences in income inequality and level of air pollution [56]. It is recommended to further elaborate this outcome using spatial epidemiology and neighbourhood effects in future. Lastly, diarrhoea was associated with a slum-level increase of the highest maternal education year, whereas *FeCo* was more prevalent in the slums where the community felt the distance between their home to the healthcare centre was the greatest. This is also true in past epidemiological studies; specifically, a qualitative study surrounding under-five child health services conducted in urban slums in Malawi found that health-seeking behaviours were negatively impacted by long distances to health facilities [57].

Most explanatory variables in our models were insignificant; with a P-value >0.05 (Delivery, birthweight, toilet type, floor type, wall type, distance to health centre and cooking fuel). Wide confidence intervals suggest that insignificant results may have been due to the small sample sizes in the analysed models. Although insignificant, it is important to address these results with regard to its wider public and global health impact, and in order to evade issues relating to underreporting bias. Our analyses therefore highlight how the explanatory variables listed above had no significant effect on the odds of developing *FeCo and diarrhoea*.

There were some limitations identified in this study, which were primarily linked to the dataset being a self-reported interview. Firstly, the dataset has a low prevalence of the outcomes, causing potential data separation (whereby observations of a particular predictor have the same outcome). This was controlled for using a Bayesian perspective generalised linear regression model, both with fixed- and random-effects, to conduct multilevel logistic modelling. Mothers’ responses regarding children’s symptoms in the last two weeks may suffer from recall bias or misinterpretation, which could have led to random misclassification and bias toward the null value. We must also note, that the variable used as a proxy for birthweight of a baby (mother’s perception) is open to issues such as measurement error; this is likely as it is subject to personal perceptions and possible systematic errors [58]. However, such perceptions from the NFHS-4 used in this study has been validated in various studies as aforementioned in Table 1 in S1 File.

Lastly, some important variables that were chosen to be included in the initial model had to be omitted at the final model, due to data limitations. For example, the Hot-deck imputation method was used to handle missing data for variables that had 50% or less missingness. This method is widely used for missing data in statistics as it reflects the real values and avoids strong parametric assumptions [39]. Multiple Imputation by Chained Equations (MICE) was also considered, however, due to MICE’s limitation on potential incompatible conditional distributions between categorical and continuous variables, it was concluded that it was not suitable for this study’s dataset [59]. It also incorporates the original dataset’s covariate information and can provide strong inferences in regression models, if imputation uncertainty is carefully considered beforehand. Variables with over 50% of values missing were excluded from the analyses.

Current literature show that this method requires a good match of donors to be able to create meaningful values. As this disadvantage is controlled better when there is a large dataset; a sample size of 1424 can be limited in providing high quality covariate variables of Missing-Not-at-Random (MNAR) NAs. This is the suggested reason on why some values showed *infinite* upper confidence intervals (values that were very low in proportion compared to other values in the categorical variable) and needed to be omitted from the final modelling analysis. It is recommended that a future study should use the MICE instead of hot-deck imputation for qualified missing variables to investigate the changes raw bias and Root Mean Squared Error (RMSE) in the dataset. This can improve the results in odds ratio and provide tighter models with higher statistical significance.

## Conclusions

This study has examined how the different social determinants of children’s health in peri-urban slums in eight cities in India impact the phenotype of RTIs (proxy: *FeCo*) and GI infections (proxy: *diarrheoa*). In sum, the study found that at an individual level, the odds of developing *FeCo* were higher if the mother thinks the child was born *smaller than average* (AOR 4.41, 1.13–17.17). It was also found that the odds of the diarrhoea outcome were lower if the child was *older* (AOR 0.97, 0.96–0.98), and at a household level; the diarrhoea outcome was found to also be lower if the household’s water source is *public tap or standpipe* (AOR 0.54, 0.31–0.96).

This study provides valuable information for future policy interventions to lower the odds of childhood morbidities in marginalised slum populations. It provides empirical evidence of the need to approach each slum cluster with different ideas, firmly rooted in the local context. Crucially, any intervention aiming to improve child health should tackle all levels, and all areas, of the social determinants of health outcomes. Moreover, the protective and risk factors identified in this study should provide a guide to state and national level policymakers to prioritise addressing to improve child health outcomes across these eight cities and serve as a template for other Indian cities. The policies should utilise a cross-sector, yet multilevel approach.

## Supporting information

S1 File Selection rationale of variables. (DOCX)
