# Supplementary material for: What are the determinants of childhood infections in India’s peri-urban slums? A case study of eight cities
Source: PLoS One. 2021 Oct 15;16(10):e0257797. doi: 10.1371/journal.pone.0257797 (PMC8519422; doi:10.1371/journal.pone.0257797)
Supplement: S1 File — (DOCX) [file pone.0257797.s001.docx]

**Supplementary Materials 1. Selection rationale of Variables**

**Table 1. Selection rationale of Variables at the Individual level**

| **Variable name** | **Rationale** |
| --- | --- |
| Sex of child | The sex-differences in virulence of infections have been observed in the past literature [21,22]. Male infants are more likely to be more susceptible to infections. Additionally, female children are less likely to leave the household, and are less likely to access medical treatment or vaccinations, than males in south-central Asia [23]. |
| Child's age in months. | As a child gets older, their immature and innate immune system starts to mature and acquire memory to fight off pathogens. Therefore, controlling for age in children when looking at the incidence of infections is significant [24,25]. |
| Birthweight (mother’s perception). | Low-birthweight children have an increased risk of contracting RTIs and GI infections. Since there is a significant incidence of home delivery (M15), a mother's opinion of the birthweight was used as a variable instead of the actual birth weight [26,27].  To note, mother’s perception of the size of her baby at birth was used as a proxy for birthweight in this study. Such perceptions are known to be dependent on cultural and ethnic factors [28]. Existing studies have examined the reliability of using this measure from the NHSF-4, and have concluded that birth weight is closely related to mother’s perception of size [29].  Other studies have also assessed the reliability in perception vs. birthweight. This was completed through comparing average weights of babies by reported sizes were examined in tests of association where birth weights were independently available [30]. |
| The month of the interview. | India faces heavy rainfall during the Monsoon season, which has associations with RTIs and GI infections if there are inadequate sanitation or water sources (related to slum environment) [31–33]. |
| Were never breastfed. | Breastfed children may have protection from infection as breast milk is known to provide passive and likely long-lasting active immunity [34]. |

**Table 2: Selection rationale of Variables at the Household level**

| **Variable name** | **Rationale** |
| --- | --- |
| Highest year of education of a mother. | Several papers have found that maternal education has positive effects on childhood health outcomes [35,36] |
| Source of drinking water. | Faecal contamination in drinking water is a major problem in LMIC urban slums, and a paper which looked into slums in Indonesia found different water sources (e.g. dug wells, refill bottled water) containing different levels of contaminants that are linked to infections [37]. |
| Type of toilet facility. | Improved toilet facilities are known to have significant health, social and economic benefits in LMIC [38]. |
| Wealth index: household has electricity; radio; television; refrigerator; telephone (landline); bicycle; motorcycle/scooter; car/truck; mosquito bed net for sleeping in the household; covered by health insurance | The wealth index in NFHS-4 presents a composite measure of a household's cumulative living standard. In addition to the index score, this study utilises different ownership markers to measure household socio-economic positions. |
| The main material of floor; wall; roof. | Ventilation and air-borne contagions, as well as associations between roof material improvement and malaria prevention, have been studied in the past. Including built environment factors will permit further investigation of the effects of improved housing materials on children’s health [39,40]. |
| Religion; Caste or tribe. | Some studies have discovered protective health measures of particular religions at the lower caste level, as well as an association of health outcomes with Scheduled Caste status and religion. Including the above variables was deemed appropriate as they may capture a lot of information about the social determinants of health in India, where the caste system officially existed until 1950 and has an important legacy today [41,42]. |
| Type of cooking fuel; Environmental tobacco smoke | Literature focusing on NFHS-2 has looked into how cooking fuels and environmental tobacco smoke are linked to children’s respiratory infections. The variables were chosen to see how the association has changed in NFHS-4 [43]. |
| Current marital status of the mother. | A study looking into single motherhood and child health in sub-Saharan Africa presented a significantly higher risk of child mortality and vulnerability in children with single mothers. This variable was chosen to investigate such associations in the Indian slum [44]. |
